# Realgar (As_4_S_4_), a traditional Chinese medicine, induces acute promyelocytic leukemia cell death via the Bcl-2/Bax/Cyt-C/AIF signaling pathway *in vitro*

**DOI:** 10.18632/aging.204281

**Published:** 2022-09-12

**Authors:** Zonghong Li, Ruiming Zhang, Xuewei Yin, Nana Li, Siyuan Cui, Teng Wang, Xing Tan, Mingyue Shen, Yun Guo, Jinxin Wang, Dadong Guo, Ruirong Xu

**Affiliations:** 1 First Clinical Medical College, Shandong University of Traditional Chinese Medicine, Jinan 250000, Shandong Province, China; 2 Department of Hematology, Linyi Hospital of Traditional Chinese Medicine, Linyi 276002, Shandong Province, China; 3 Shanghai Municipal Hospital of Traditional Chinese Medicine, Shanghai University of Traditional Chinese Medicine, Shanghai 200071, China; 4 Department of Hematology, The Affiliated Hospital of Shandong University of Traditional Chinese Medicine, Jinan 250000, Shandong Province, China; 5 College of Pharmacy, Shanxi Medical University, Taiyuan 030001, Shanxi Province, China; 6 Center for Reproductive Medicine, Cheeloo College of Medicine, Shandong University, Jinan 250000, Shandong Province, China; 7 Shandong Provincial Key Laboratory of Integrated Traditional Chinese and Western Medicine for Prevention and Therapy of Ocular Diseases, Shandong Academy of Eye Disease Prevention and Therapy, Affiliated Eye Hospital of Shandong University of Traditional Chinese Medicine, Jinan 250000, Shandong Province, China

**Keywords:** realgar, APL, Bcl-2, Bax, Cyt-C, AIF

## Abstract

Acute promyelocytic leukemia (APL) is a specific subtype of acute myelogenous leukemia (AML) characterized by the proliferation of abnormal promyelocytes. Realgar, a Chinese medicine containing arsenic, can be taken orally. Traditional Chinese medicine physicians have employed realgar to treat APL for over a thousand years. Therefore, realgar may be a promising candidate for the treatment of APL. Nevertheless, the underlying mechanism behind realgar therapy is largely unclear. The present study aimed to investigate the effect of realgar on cell death in the APL cell line (NB4) *in vitro* and to elucidate the underlying mechanism. In this study, after APL cells were treated with different concentrations of realgar, the cell survival rate, apoptotic assay, morphological changes, ATP levels and cell cycle arrest were assessed. The expression of Bcl-2, Bax, Cytochrome C (Cyt-C) and apoptosis-inducing factor (AIF) at the mRNA and protein levels were also measured by immunofluorescence, quantitative PCR (qPCR) and Western blotting. We found that realgar could significantly inhibit APL cell proliferation and cell death in a time- and dose-dependent manner. Realgar effectively decreased the ATP levels in APL cells. Realgar also induced APL cell cycle arrest at the S and G2/M phases. Following realgar treatment, the mRNA and protein levels of Bcl-2 were significantly downregulated, whereas the levels of Bax, Cyt-C, and AIF were significantly upregulated. In summary, realgar can induce APL cell death via the Bcl-2/Bax/Cyt-C/AIF signaling pathway, suggesting that realgar may be an effective therapeutic for APL.

## INTRODUCTION

Acute promyelocytic leukemia (APL), which is caused by a balanced translocation t(15;17)(q22;q12) that results in the fusion transcript PML-RARA, is a rare type of acute myeloid leukemia (AML) that accounts for 10–15% of newly diagnosed cases [1]. It is induced by aberrant promyelocytes in the bone marrow proliferating malignantly and failing to differentiate, preventing normal white blood cell generation [2]. Clinical manifestations of APL mainly include anemia, hemorrhage, infection, hepatosplenomegaly, lymphadenopathy, and bone pain [3, 4]. Among them, bleeding tendency and thrombotic events are the most common causes of mortality in APL patients [5–8]. APL is a kind of leukemia with a dangerous prognosis in the early phase [9]. With all-trans retinoic acid (ATRA) and arsenic trioxide (ATO) development, APL has progressed from a fatal illness to one of the most treatable cancers [10].

In 1973, anthracycline monotherapy was successfully utilized in APL for the first time [11]. In the mid-1980s, ATRA was utilized in patients with APL, and it had a high response rate [12, 13]. ATO is the main effective component of the traditional Chinese medicine arsenic. Chinese scholars first used it in the treatment of APL and achieved a remarkable curative effect. At present, ATO is considered to be one of the most successful medications for the treatment of APL. However, treatment refractoriness and side effects have been noted in the clinical environment. Anthracyclines can cause severe cardiotoxicity, and ATRA syndrome (RAS) is the most serious complication of ATRA induction in the treatment of APL [14]. Moreover, prolonged ATO exposure can easily cause arsenic poisoning, including skin lesions, neurological effects, diabetes and bladder cancer [15]. Furthermore, patients receiving ATO must be hospitalized for intravenous injection, which is inconvenient and not cost-effective. Consequently, an oral drug with comparable efficacy, fewer side effects, and improved patient compliance should be developed in order to offer patients an improved quality of life as well as consolidation and maintenance therapy.

Realgar, a traditional Chinese medicine (TCM), has been applied for more than 1800 years by traditional Chinese doctors [16]. Similar to ATO, realgar is also a kind of mined ore that contains arsenic. Currently, it is widely accepted that the benefit and risk of arsenic are solely dependent on the chemical forms of arsenic rather than the arsenic content alone [17]. Realgar’s monoclinic crystal structure is cradle-type with an octatomic ring spatially and includes approximately 90% As_4_S_4_, of which the crystalline form is α-As_4_S_4_. Previous studies have shown that realgar is less toxic than ATO [18, 19]. Since 1963, it has been included in the Chinese Pharmacopoeia (ChP). Employed either externally or orally, it is effective in treating a variety of exterior and interior disorders [20, 21]. In recent years, realgar has been shown to have anticancer properties [22–27]. Similar clinical observations also support realgar as a drug for various types of hematological malignancies [28]. In China, realgar has been utilized as a single treatment for APL [29]. Chen et al. revealed that realgar can induce apoptosis and differentiation in APL cells [30]. In a clinical study including 129 APL patients, the results showed that oral realgar treatment can achieve high rates of complete remission, tolerable side effects and long-term disease-free survival [31]. In addition, realgar, which can be administered orally, is preferable to ATO, which can only be administered by injection. Because oral administration is more conducive to consolidating and maintaining therapy, which can improve the compliance and quality of life of APL patients, realgar is a promising candidate worthy of further exploration for the treatment of APL.

Despite the fact that realgar has been used to treat APL in China for many years, its molecular mechanism remains unclear. The purpose of this study was to investigate the effect of realgar on the death of the APL cell line NB4 and to clarify the underlying mechanism. In this study, APL cells were treated with different concentrations of realgar, and cell viability, apoptosis, morphological changes, ATP levels and cell cycle arrest were measured, and we evaluated, for the first time, the expression of Bcl-2, Bax, Cyt-C and AIF at the mRNA and protein levels following realgar treatment. Our study is helpful for understanding the potential mechanism of realgar in the treatment of APL.

## MATERIALS AND METHODS

### Realgar and preparation of realgar solutions

Realgar containing arsenic sulfide (As_4_S_4_) with a purity of 93.4% was obtained from Hubei Pharmaceutical Co., Ltd. (Wuhan, China). In the current investigation, 3 g of realgar was sieved through a 500 mesh sieve, added to distilled water to a volume of 300 mL, and mixed for 30 minutes using a magnetic stirrer. It was then put into an M-110EH high-pressure microjet granulator (ATSE Nanotechnology Ltd., Suzhou, China) for 10 cycles at 30,000 rpm, followed by 30 minutes of ultrasonic vibration. After grinding, the realgar nanoparticles were spherical and uniform in size, and the average diameter measured by a Malvern Zetasizer Nano-ZS (Malvern Instruments, Britain) was approximately 72.63 nm. The details of realgar nanoparticles Dynamic Light Scattering (DLS) and particle size are shown in Supplementary Figure 1. After degassing using a 0.22 μm micropore screen, the concentration of total arsenic was determined by inductively coupled plasma-atomic emission spectrometry (ICP–AES). Finally, stock solutions of realgar (1000 μg/mL) were prepared in RPMI 1640 medium, and stored airtight and light-shielded at 4°C.

### Cells and cell culture

The APL cell line (NB4, obtained from ATCC) was cultured in RPMI-1640 media (Solarbio, Beijing, China) supplemented with 10% fetal bovine serum (HyClone, Logan, UT, USA), 100 μg/mL streptomycin (Sigma, St. Louis, MO, USA) and 100 U/mL penicillin (Sigma, St. Louis, MO, USA). APL cells were cultured at 37°C in an incubator with 5% CO_2_ and 95% air. Cell numbers were determined by an automated cell counter (Muse; Merck Millipore, Billerica, MA, USA). All experiments were performed on an ultraclean bench.

### Cell counting kit-8 (CCK-8) assay

APL cells in the logarithmic growth phase were cultured in 96-well plates (Thermo Fisher Scientific Ltd., China). In brief, 100 μL of the cell solutions (cell density of 8.0 × 10^4^/mL) were added to each well of the 96-well plate. Next, realgar solutions at concentrations of 0, 12.5, 25.0, 50.0 and 75.0 μg/mL (the concentration of arsenic contained corresponds to 0.3, 0.6, 1.2 and 1.5 μg/mL, respectively) were added to each well. Then, the cells were cultivated for 24, 48, and 72 hours in an incubator at 37°C with 5% CO_2_ and water saturation humidity. Finally, each well’s optical density (OD) at 450 nm was determined using a microplate reader (BioTek Instruments, Winooski, VT, USA). The experiment was carried out three times independently. Furthermore, 50% inhibitory concentration (IC50) was the concentration of realgar required to inhibit the growth of APL cells by 50%.

### Cell morphology

NB4 cells were seeded in 6-well plates at a density of 1.2 × 10^5^ cells/well and then exposed to different concentrations (0, 12.5, 25.0, 50.0 and 75.0 μg/mL) of realgar (final volume: 3 mL) for 24 h. At the indicated times, morphological alterations were observed using a light field microscope (IX71, Olympus, Japan).

### Apoptosis/necrosis analysis

NB4 cells were plated at a density of 1.2 × 10^5^ cells/well in 6-well plates, and then the cells were exposed to various concentrations (i.e., 0, 12.5, 25.0, 50.0, 75.0 μg/mL) of realgar (final volume: 3 mL) for 24 h. Next, the cells were collected at the specified time, gently resuspended by adding 195 μL Annexin V-FITC Binding Buffer (Beyotime, China), incubated with 5 μL Annexin V-FITC and 10 μL PI staining solution for 20 minutes at room temperature (20−25°C) and protected from light, and placed on ice for detection by flow cytometry (BD FACSVerse, USA). Annexin V-positive and PI-negative cells indicated the early stages of apoptosis, whereas both Annexin V- and PI-positive cells indicated the late stages of apoptosis.

### Cell cycle analysis

Cell cycle arrest analysis was carried out by flow cytometry after APL cells were exposed to different concentrations (0, 12.5, 25.0, 50.0 and 75.0 μg/mL) of realgar and stained with the DNA-specific dye ethidium bromide. In short, 1.2 × 10^5^ cells were seeded in a six-well tissue culture plate, and various concentrations (0, 12.5, 25.0, 50.0 and 75.0 μg/mL) of realgar were added and incubated for an additional 24 h. Next, the cells were collected, washed with 1× PBS, and centrifuged. Then, the obtained cells were rewashed in PBS, fixed in 70% ice-cold ethanol and kept at −20°C. Finally, the cells were rewashed with PBS and stained with ethidium bromide, followed by analysis by flow cytometry (BD FACSVerse, USA).

### Determination of ATP level

To investigate the effect of realgar on the production of ATP, the ATP content was evaluated using an ATP assay kit (Solarbio, BC0300, China). Briefly, APL cells were cultured with various concentrations of realgar for 24 hours (i.e., 0, 12.5, 25.0, 50.0, 75.0 μg/mL), and then ATP-releasing reagent was added and ultrasonicated on ice for 10 minutes, followed by centrifugation at 12,000 g at 4°C for 10 minutes to collect the supernatants. Finally, a MultiMode Microplate Reader (BioTek Instruments, Winooski, VT, USA) was used to determine the results.

### Immunofluorescence analysis

NB4 cells were treated with realgar under optimal conditions (75.0 μg/mL, 72 h) as determined by the CCK-8 assay. Following treatment, the cells were spread onto slides, fixed with 4% paraformaldehyde for 20 minutes at 4°C, and permeabilized in 0.1% Triton for 10 minutes at room temperature. Then, the cells were incubated with 10% goat serum (Beyotime Institute of Biotechnology) for 30 minutes at room temperature before the primary antibody reaction. After that, the slides were treated for 8 hours at 4°C with rabbit anti-human polyclonal antibody against RARa (1:200; Santa Cruz Biotechnology, Inc.), followed by FITC-conjugated goat anti-rabbit IgG (1:200, Zhongshan Goldenbridge Biotechnology Co., Ltd.) at room temperature for 1 h. Next, microscopy was used to observe the cells after counterstaining with DAPI (1 μg/mL, 5 min). A fluorescence microscope (×400; Nikon Corp, Tokyo, Japan) and a confocal laser scanning microscope (×400; Leica Microsystems GmbH, Wetzlar, Germany) were used to observe the cells.

### Quantitative reverse transcription PCR (qPCR)

We used qPCR to investigate the expression of Bcl-2, Bax, Cytochrome C (Cyt-C), and apoptosis-inducing factor (AIF) mRNA levels in cells treated with different concentrations of realgar. In brief, APL cells (1.2 × 10^5^/well) were seeded in six-well plates and treated for 24, 48 and 72 h with various concentrations (0, 12.5, 25.0, 50.0, and 75.0 μg/mL) of realgar solution. Then, the cells were collected, and total RNA was extracted by an RNA Tissue/Cell Rapid Extraction Kit (Shandong Sparkjade Science Co., Ltd., China). Total RNA was quantified by a microspectrophotometer, followed by generation of single-stranded cDNA from 600 ng total RNA. Furthermore, qPCR was performed in order to measure the gene level using cDNA synthesized by reverse transcription. Table 1 lists the target-specific primers, and the PCR program was configured as follows: 94°C for 3 min, then 94°C for 10 s, then 40 cycles of 94°C for 5 s, and 60°C for 34 s. After normalization to the relevant endogenous GAPDH control, the fold changes were determined using the 2^−ΔΔCt^ technique.

**Table 1:** Primer sequences for the target genes determined by quantitative PCR.

**ID**	**Primer**	**Sequences (5′ to 3′)**
AIF	Forward	GTG ATT TGG GCC CCG ATG TT
	Reverse	GGG GTG CTG GGA GGA ATA GT
Cyt-C	Forward	TCC GGC TGG TAG TAG TTC CG
	Reverse	TTC CGC CAT GGT GCT GAA TC
Bax	Forward	CGG GTT GTC GCC CTT TTC TA
	Reverse	GCT CCC GGA GGA AGT CCA AT
Bcl-2	Forward	TGA GTT CGG TGG GGT CAT GT
	Reverse	TCA GTC ATC CAC AGG GCG AT
GAPDH	Forward	TGC ACC ACC AAC TGC TTA GC
	Reverse	GGC ATG GAC TGT GGT CAT GAG

### Western blotting

Total proteins were extracted from the realgar-treated NB4 cells, and the protein concentration was measured using a BCA Test Kit (Beyotime, China). The extracted proteins were denatured with reducing sample buffer at 100°C for 7 min. Then, 40 μg of protein was loaded onto a 10% polyacrylamide gel. Proteins were separated (30 min at 80 V, then 120 V until completed) with Tris-HCl running buffer in an electrophoresis apparatus (BioRad, Hercules, CA, USA). Then, the proteins were transferred (90 min, 100 V) from the acrylamide gel to a polyvinylidene fluoride (PVDF) membrane. Blots were blocked with 5% bovine serum albumin (BSA) for 1 h, followed by incubation with monoclonal anti-Bax (1:1000, ab32503, Abcam, USA), Bcl-2 (1:1000, ab32124, Abcam, USA), AIF (1:1000, ab32516, Abcam, USA), and Cyt-C (1:5000, ab133504, Abcam, USA) antibodies at 4°C overnight. Subsequently, the membranes were washed frequently with Tris-buffered saline Tween (TBS-T; 5 min, 3 times), followed by incubation with goat anti-rabbit IgG antibody (1:20,000) at room temperature for 1 h. After repeated washings with TBS-T, the blots were treated with a chemiluminescent detection kit (Thermo Fisher Scientific, China). The internal reference was represented by an antibody against actin. The quality of the protein was assessed using a Typhoon PhosphorImager (GE Healthcare, Piscataway, NJ, USA).

### Statistical analysis

All experiments were performed at least three times independently, and the data are expressed as the mean ± the standard deviation. For the significance test, one-way analyses of variance and post hoc methods depending on Newman–Keuls tests were applied. *P* < 0.05 was considered to be significant. SPSS 25.0 was used to analyze the data.

## RESULTS

### Realgar inhibits NB4 cell proliferation

**Figure 1:**
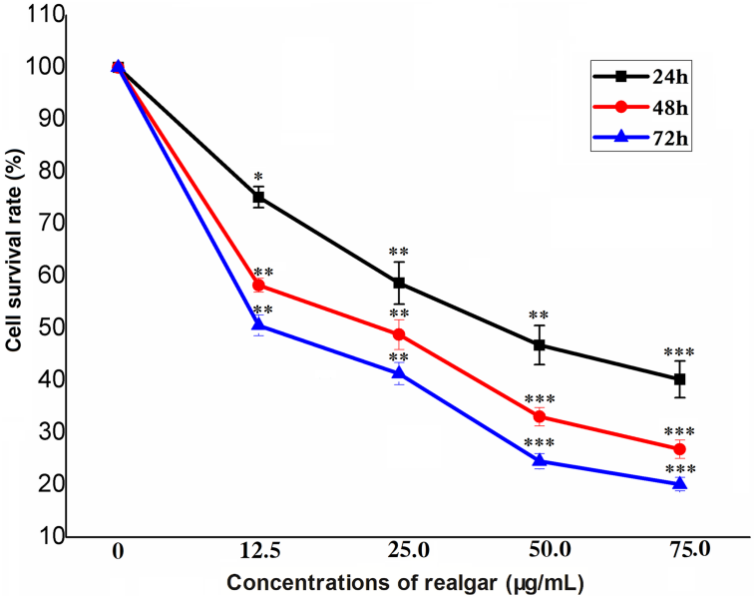
**Cell survival after NB4 cells were exposed to different concentrations of realgar.** Cell survival was evaluated using a CCK-8 assay after treatment with different concentrations of realgar for 24 h, 48 h or 72 h. *N* = 3.

The dose- and time-dependent responses of NB4 cells to realgar are shown in Figure 1. When the concentration of realgar was increased, the survival rate of NB4 cells was reduced in a time-dependent manner. The IC50 values of realgar in NB4 cells were 48.0, 27.0, and 14.0 μg/mL after 24, 48 and 72 h, respectively.

### Change in cell morphology

**Figure 2:**
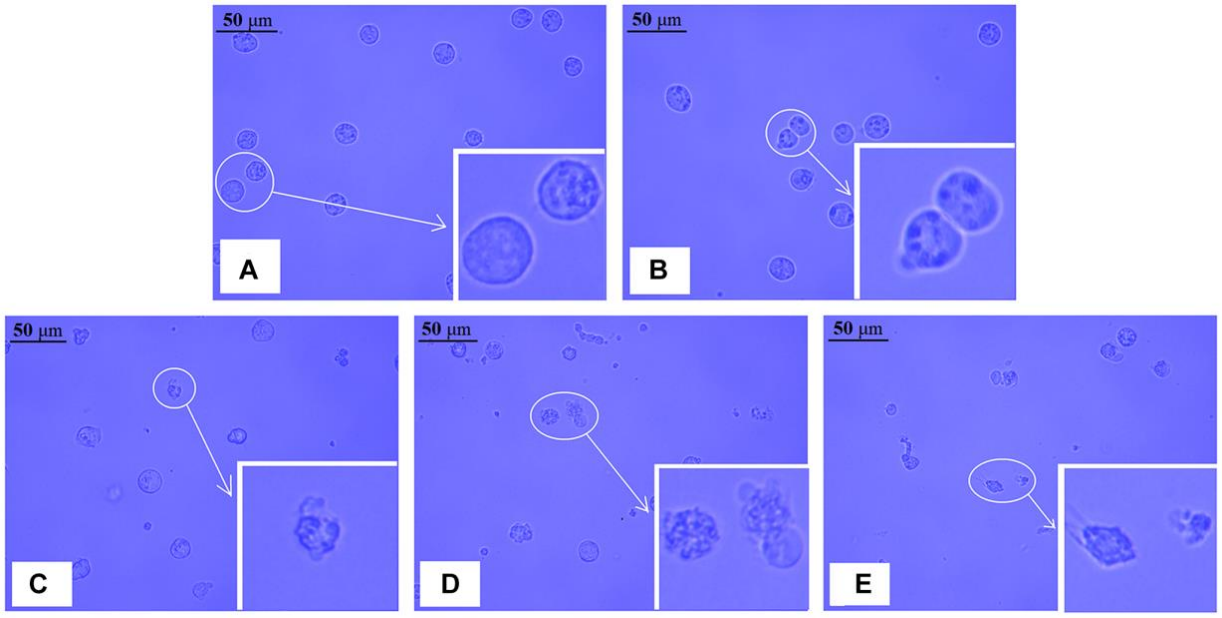
**Morphological changes in NB4 cells after exposure to different concentrations of realgar for 24 h.** The cells were treated with different concentrations of realgar for 24 h, and typical images were captured by a light field microscope. (**A**) Control cells; (**B**) cells exposed to 12.5 μg/ml of realgar; (**C**) cells exposed to 25.0 μg/ml of realgar; (**D**) cells exposed to 50.0 μg/ml of realgar; and (**E**) cells exposed to 75.0 μg/ml of realgar. Inset, magnified cells.

As illustrated in (Figure 2A–2E), we found that the number of intact NB4 cells decreased, while the number of damaged cells increased, including cell contraction, nuclear condensation, and debris. The higher the concentration of realgar, the more significant the alteration of cell morphology. According to these results, realgar has a significant cytotoxic impact on NB4 cells.

### Annexin V-FITC/PI staining analysis

**Figure 3:**
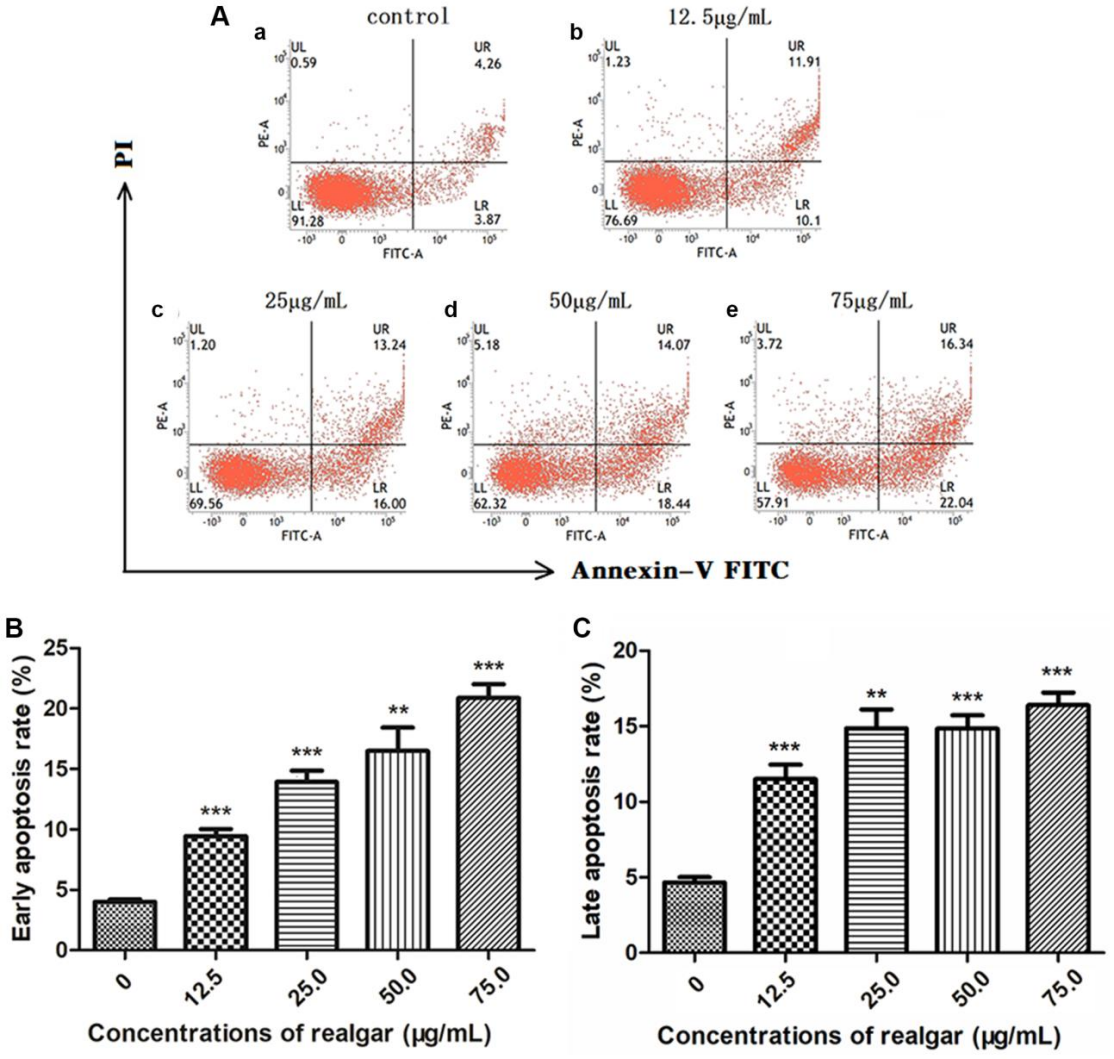
**Analysis of apoptosis/necrosis of NB4 cells after exposure to different concentrations of realgar for 24 h.** The cells were treated with different concentrations of realgar (0, 12.5, 25.0, 50.0 and 75.0 μg/ml) for 24 h, followed by staining with the Annexin V-FITC/PI apoptosis staining kit. Apoptosis/necrosis analysis was performed by flow cytometry. (**A**) (**a**) Untreated cells; (**b**) cells treated with 12.5 μg/ml of realgar; (**c**) cells treated with 25.0 μg/ml of realgar; (**d**) cells treated with 50.0 μg/ml of realgar; and (**e**) cells treated with 75.0 μg/ml of realgar. In addition, histogram analysis was performed on early apoptosis (**B**) and late apoptosis (**C**). Data were obtained from three independent experiments and are presented as the mean ± S.D. *N* = 3.

Annexin-V-FITC and PI double staining was used to distinguish between live cells, early apoptotic cells, and late apoptotic/necrotic cells after realgar treatment of NB4 cells (Figure 3A). We observed that the amount of early apoptosis and late apoptosis/necrosis in NB4 cells was noticeably increased in a concentration-dependent manner after being cultured with various concentrations of realgar for 24 hours (Figure 3B and 3C). The results indicate that apoptosis can be easily induced in NB4 cells by realgar treatment.

### Effect of realgar treatment on cell cycle arrest

**Figure 4:**
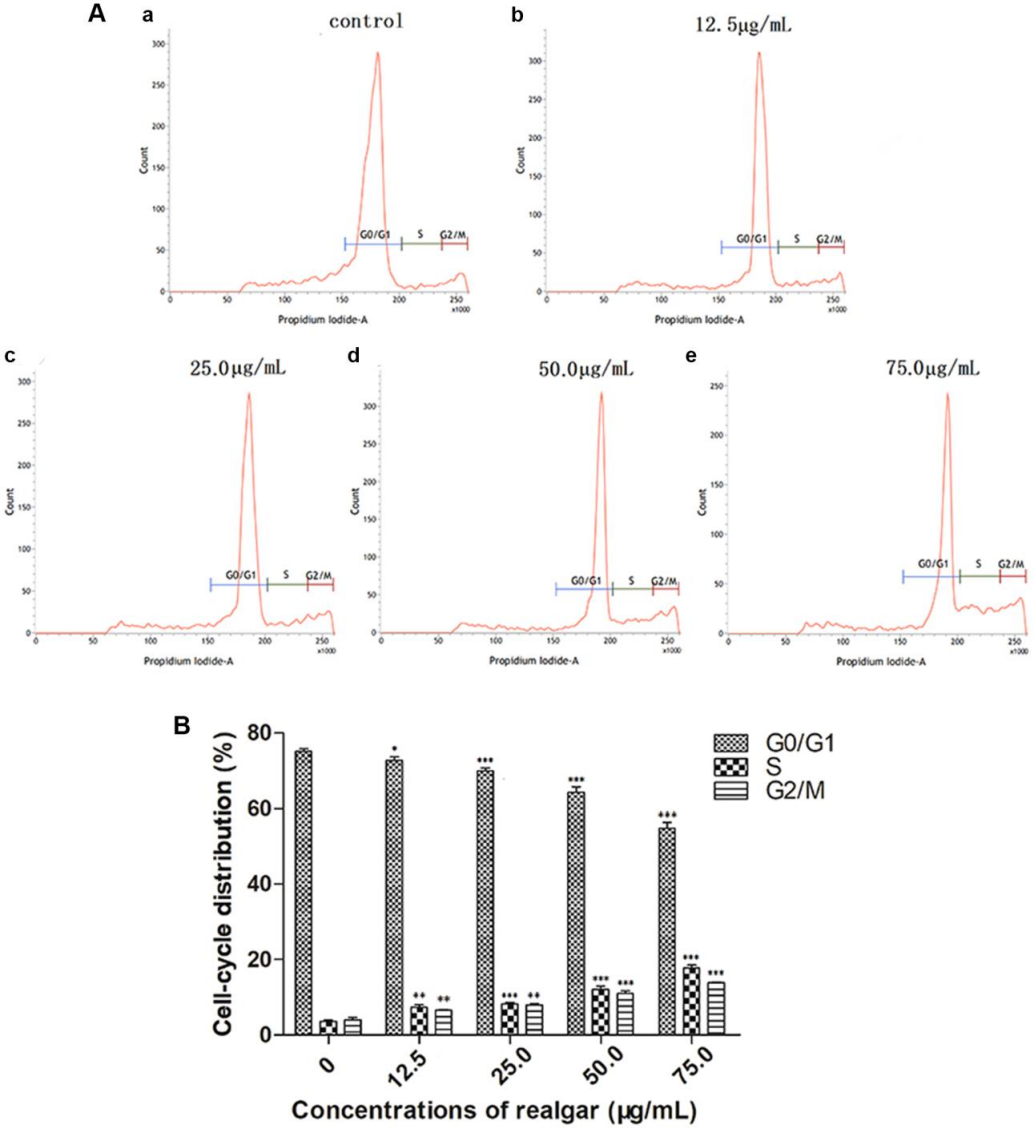
**Effect of realgar on APL cell cycle arrest.** (**A**) (**a**) Control, (**b**) 12.5 μg/mL, (**c**) 25.0 μg/mL, (**d**) 50.0 μg/mL, and (**e**) 75.0 μg/ml. The statistical data were generated by flow cytometry software and plotted as generated. (**B**) Histogram analysis of cell cycle phase distributions of NB4 cells after treatments with 0, 12.5, 25.0, 50.0 and 75.0 μg/mL of realgar. Data were obtained from three independent experiments and are presented as the mean ± SD. ^*^P < 0.05, ^**^P < 0.01 and ^***^P < 0.001.

To verify whether realgar suppressed the proliferation of NB4 cells by triggering cell cycle arrest, we evaluated the change in the cell cycle distribution after NB4 cells were treated with realgar. Figure 4A shows that realgar caused S and G2/M phase accumulation, and correspondingly, G0/G1 phase was decreased in NB4 cells. After exposure to various concentrations of realgar for 24 h, it was discovered that the S phase increased from 3.69% to 7.43%, 8.30%, 12.07%, and 17.84%, and the G2/M phase increased from 4.12% to 6.54%, 8.02%, 11.04%, and 13.86%, and there was a statistically significant difference compared to the control samples (Figure 4B). These results suggested that realgar can induce APL cell cycle arrest to inhibit proliferation in a dose-dependent manner.

### ATP levels

**Figure 5:**
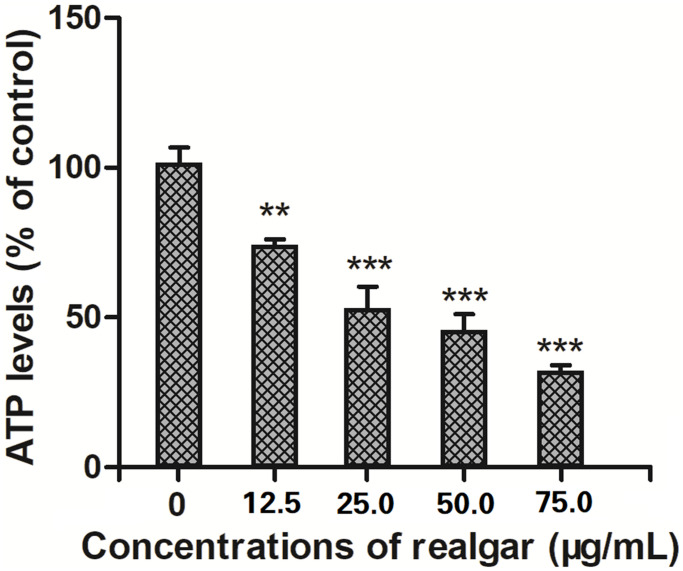
**Effect of different concentrations of realgar on ATP generation in APL cells for 24 h.** The cells were treated with different concentrations of realgar (0, 12.5, 25.0, 50.0 and 75.0 μg/ml) for 24 h. Then, the ATP levels were measured using a commercial ATP assay kit according to the manufacturer's instructions. Data were obtained from three independent experiments and are presented as the mean ± SD. ^**^P < 0.01 and ^***^P < 0.001.

After 24 hours of treatment with different concentrations of realgar for 24 hours, the ATP levels in NB4 cells declined from 100% to 76.409%, 54.008%, and 48.825% (Figure 5). The results showed that ATP generation by NB4 cells was drastically inhibited following exposure to realgar.

### Bcl-2, Bax, Cyt-C, and AIF gene expression

**Figure 6:**
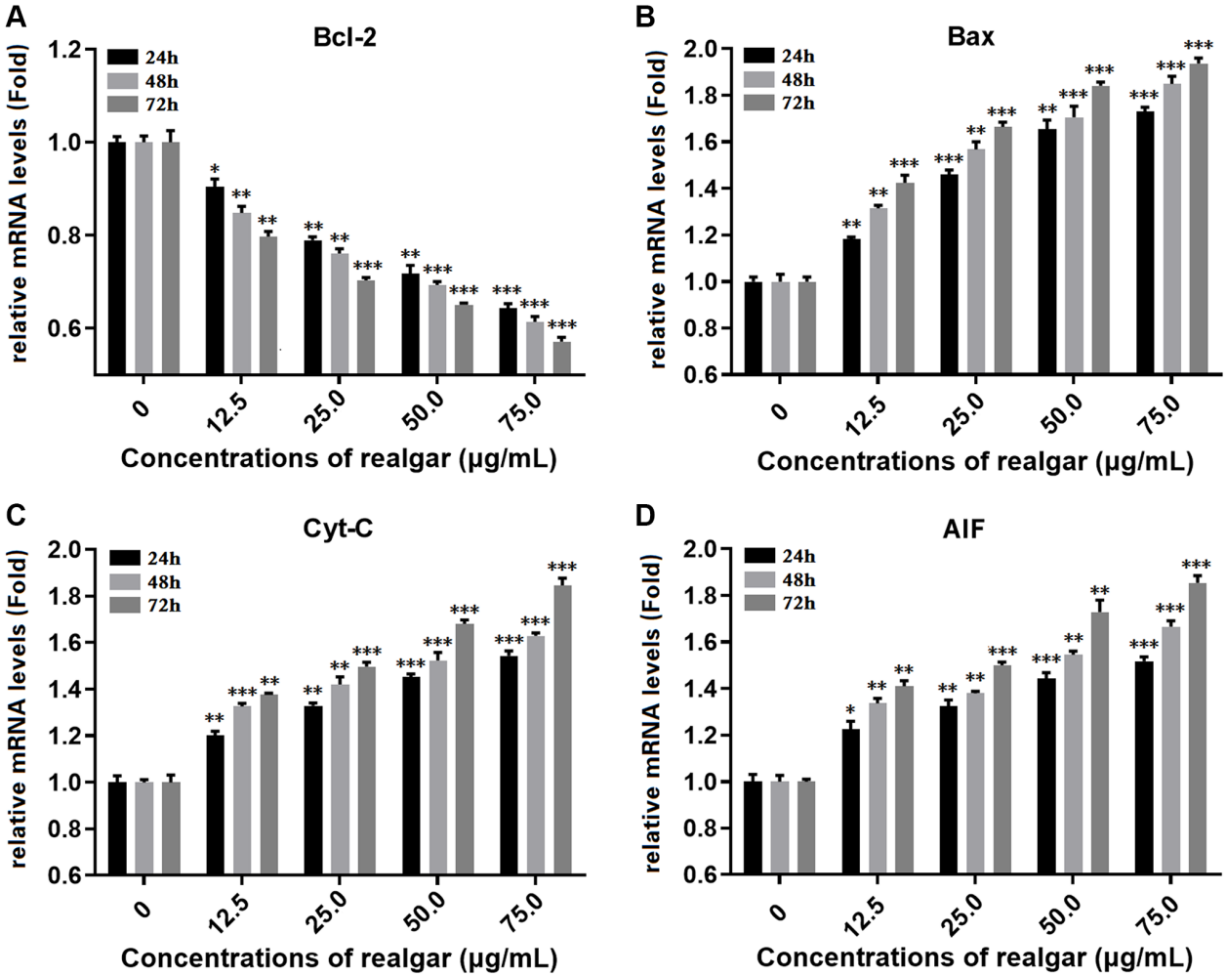
**Measurement of the gene expression of Bcl-2, Bax, Cyt-C and AIF.** NB4 cells were treated with different concentrations of realgar (0, 12.5, 25.0, 50.0 and 75.0 μg/ml) for 24, 48 and 72 h, and the mRNA expression levels of Bcl-2, Bax, Cyt-C and AIF were compared to those in untreated cells. (**A**) Bcl-2 mRNA level; (**B**) Bax mRNA level; (**C**) Cyt-C mRNA level and (**D**) AIF mRNA level. The results are expressed as the mean ± S.D. (standard deviation) of three independent experiments. ^*^P < 0.05, ^**^P < 0.01 and ^***^P < 0.001.

In this study, the impact of different concentrations of realgar on the levels of Bcl-2, Bax, Cyt-C, and AIF gene expression in NB4 cells was investigated using qPCR. As shown in (Figure 6A–6D), the expression levels of the Bcl-2, Bax, Cyt-C and AIF genes were differentially regulated after the cells were treated with realgar. We observed that the level of Bcl-2 in NB4 cells decreased in a dose dependent manner to 0.849-, 0.751-, 0.687- and 0.609-fold compared with the control samples after exposure to realgar, whereas Bax increased to 1.307-, 1.564-, 1.733- and 1.838-fold, Cyt-C increased to 1.301-, 1.415-, 1.552- and 1.672-fold, and AIF increased to 1.324-, 1.401-, 1.572- and 1.679-fold. These findings demonstrate that realgar treatment can cause NB4 cell death.

### Bcl-2, Bax, Cyt-C, and AIF protein expression

**Figure 7:**
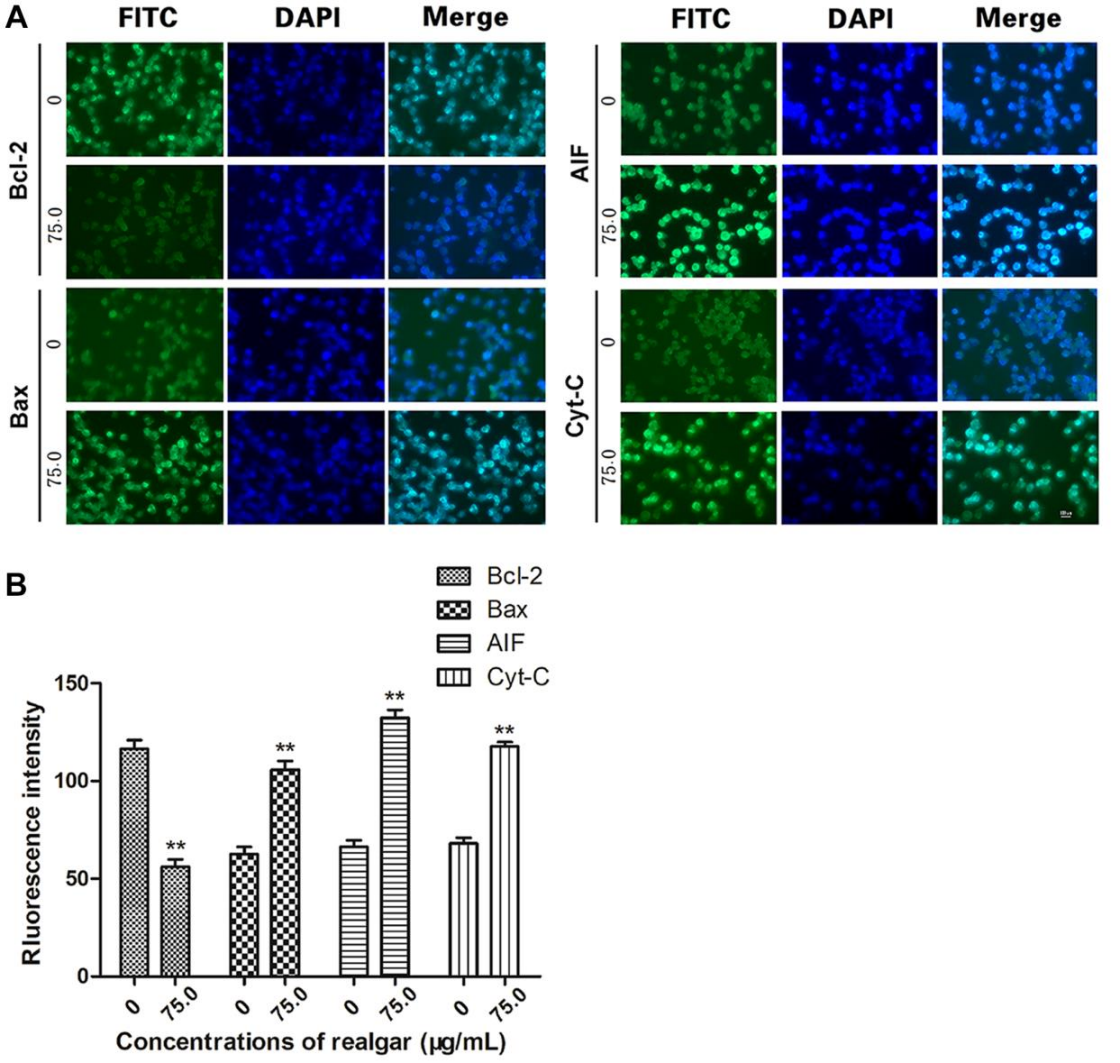
**Determination of the target protein levels.** NB4 cells were treated with or without realgar (75.0 μg/ml) for 72 h. The levels of Bcl-2, Bax, Cyt-C, and AIF were measured using immunofluorescence. (**A**) Cell nuclei were stained with 4',6-diamidino-2-phenylindole (DAPI; blue). (**B**) The fluorescence intensity of each protein is shown. Original magnification, × ^**^P < 0.01.

Typically, deregulated cell proliferation and cell apoptosis are intimately coupled. Therefore, we evaluated the effect of realgar on cell apoptosis by evaluating the expression of apoptosis-associated proteins in NB4 cells upon realgar stimulation. Based on the cell proliferation data, the optimal concentration and stimulation time (75 μg/ml, 72 h) were used in this experiment. The results of the immunoblotting analysis showed that realgar treatment clearly downregulated the expression of the antiapoptotic protein Bcl-2 and promoted the expression of the proapoptotic protein Bax. In addition, the levels of Cyt-C and AIF were also elevated (Figure 7A and 7B).

**Figure 8:**
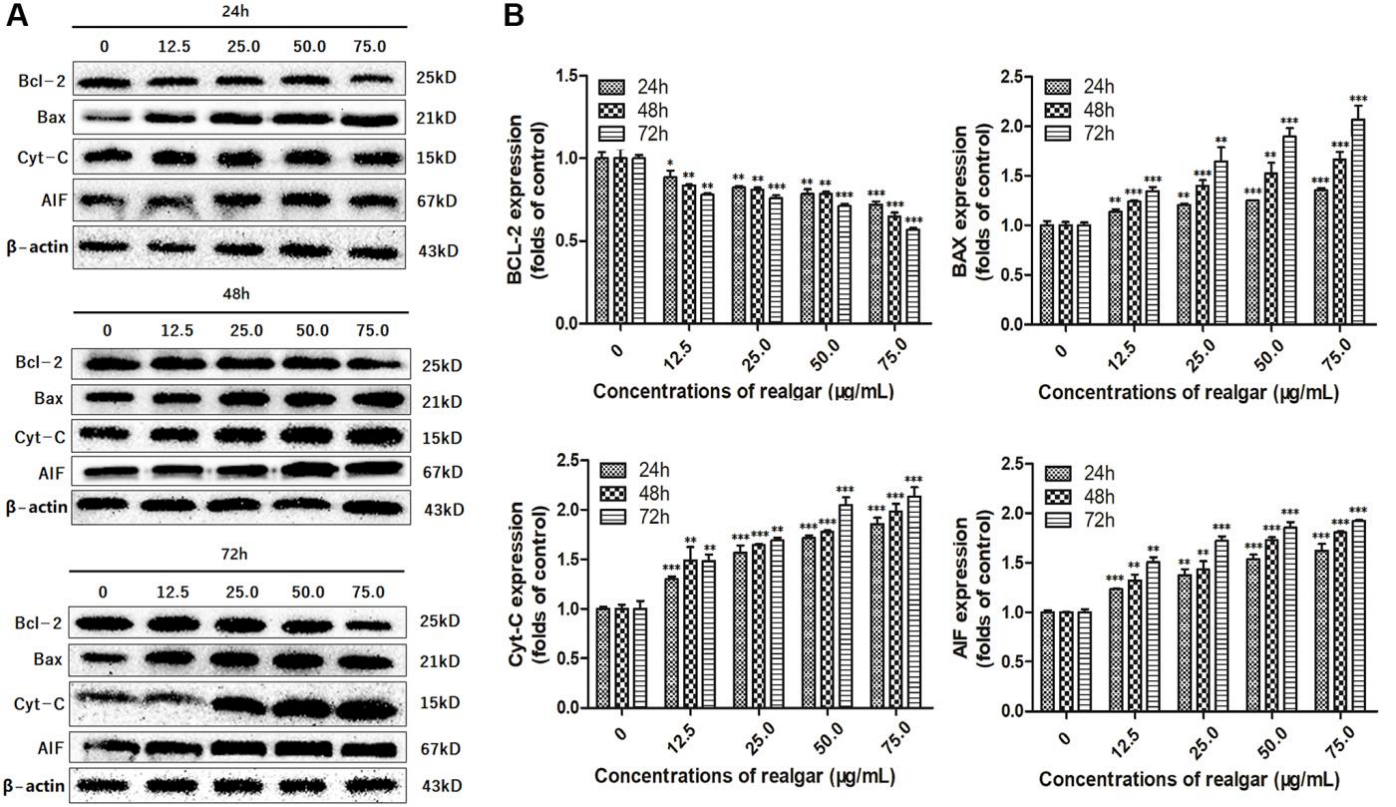
**Evaluation of the Bcl-2, Bax, Cyt-C and AIF protein levels.** Western blot analysis was performed in order to determine the Bcl-2, Bax, Cyt-C and AIF protein levels in NB4 cells after treatment with different concentrations of realgar (0, 12.5, 25.0, 50.0 and 75.0 μg/ml, respectively) for 24, 48 and 72 h, and three independent experiments were repeated. (**A**) Bcl-2, Bax, Cyt-C and AIF protein levels; (**B**) Histogram analysis of the target protein expression levels in NB4 cells. ^*^P < 0.05, ^**^P < 0.01, and ^***^P < 0.001 vs. relevant control samples.

To further verify this outcome, we performed western blotting analysis (Figure 8A). The results also showed that the level of Bcl-2 was elevated upon realgar stimulation, and in contrast, the expression of Bax, Cyt-C and AIF was decreased (Figure 8A and 8B). This finding indicates that the apoptotic pathway mediated by mitochondria in NB4 cells may be triggered by realgar exposure, implying that realgar-induced apoptosis in NB4 cells could be related to the Bcl-2, Bax, Cyt-C, and AIF signaling pathways.

## DISCUSSION

For more than 1500 years, traditional Chinese doctors have employed realgar, a traditional Chinese medicine, to treat hematological malignancies by oral administration [18, 32]. Employed either externally or orally, it has a significant therapeutic effect on carbuncles, lumps, snake bites, furuncles, parasitoses, scrofula and syphilis, among other external and interior ailments [20, 21]. Realgar is less toxic than ATO and can be administered orally. Numerous clinical studies have demonstrated that oral realgar alone or in combination with other drugs has a good therapeutic effect for APL patients [33–35]. Therefore, realgar is regarded as a promising therapeutic candidate and is worthy of further exploitation in the treatment of APL. Nevertheless, the underlying mechanism behind realgar therapy is largely unclear. In this study, we evaluated the effects of realgar on APL using the APL cell line NB4. We demonstrated that realgar has a cytotoxic impact on APL cells, inducing them to die. We hypothesized that the mechanism of realgar-induced APL cell death may be related to mitochondria-mediated apoptosis.

A CCK8 assay was used to analyze the inhibitory impact of realgar on cell proliferation. We observed that by increasing the concentration of realgar cultured with the cells and lengthening the culture period, APL cell viability gradually decreased (Figure 1). Chen et al. found that after treatment with realgar in an MTT assay, ATRA-sensitive NB4 and ATRA-resistant MR2 cells were significantly inhibited in a dose- and time-dependent manner [30]. This result is similar to our findings, indicating that realgar has a cytotoxic effect on tumor cells.

At present, apoptosis is generally thought to be the most common mechanism of cell death [36]. We found that the morphology of APL cells was altered dramatically after treatment with realgar for 24 hours. The greater the concentration of realgar used to treat APL cells, the more severe the morphological alterations (Figure 2). These results are also in agreement with our Annexin V-FITC/PI staining results (Figure 3). AIF is a protein with apoptosis-inducing activity that is located in the membrane space of mitochondria. When cells are stimulated by apoptosis, AIF molecules are released from mitochondria to the cytoplasm, then translocated to the nucleus, and combined with chromosomal DNA to agglutinate around the chromosomal nucleus and break DNA into large fragments of approximately 50 Kb. DNA damage can cause the regular processes of cells to be disrupted, eventually leading to death [37]. As a consequence, we can deduce that realgar may cause APL cells (NB4) to die by breaking DNA chains.

Cell cycle arrest has become one of the most successful cancer therapies after years of study into the cell cycle and its role in cancer development [38]. Flow cytometry analysis was performed in APL cells following treatment with realgar to determine cell cycle arrest caused by realgar. According to this study, realgar arrests the APL cell cycle in the S and G2/M phases in a concentration-dependent manner (Figure 4). Our findings support the hypothesis that realgar can cause cell cycle arrest in APL cells. Since the cell cycle-arrested cells could not enter the mitotic phase, realgar may be a potential therapy for preventing APL cell division. However, Ye et al. explored the effect of realgar on another APL cell line, HL-60, and showed that realgar-induced membrane toxicity may play an important role in the induction of apoptosis in APL cells [39].

Mitochondria are vital to cell survival. They not only produce ROS but also generate ATP through the oxidative phosphorylation system. In pathological situations, the malfunctioning of mitochondria results in ATP exhaustion [40]. We investigated the influence of realgar on the mitochondrial function of APL cells and showed that the amount of ATP produced decreased as the concentration of realgar increased (Figure 5), indicating that realgar can impact mitochondrial activity in APL cells. Based on these observations, we infer that realgar disrupts mitochondrial homeostasis, induces mitochondrial malfunction, and activates the mitochondria-mediated apoptotic signaling pathway, resulting in APL cell death. In contrast to our findings, Hai Y et al. discovered that realgar triggered NB4 cell differentiation by partly degrading PML/RARa via the ubiquitin–proteasome pathway [22].

Apoptosis, also called programmed cell death, is a kind of cell death that is vital for maintaining homeostasis [41]. It has been demonstrated that various proteins are involved in apoptosis. Bcl-2 family proteins, as important regulators of cell apoptosis, are mainly located in mitochondria and regulate cell apoptosis by influencing mitochondrial function [42]. Within the Bcl-2 family, Bcl-2 protein inhibits cell apoptosis by regulating the integrity of mitochondria and endoplasmic reticulum, promoting the release of Cyt-C and the activity of caspases [43]. In addition, Bcl-2 can integrate with other apoptosis-promoting proteins, such as Bax, and inhibit the function of Bax [44]. In the context of cellular stress, the Bcl-2/Bax expression ratio has been suggested to indicate cell survival. Over the last decade, evidence has emerged that Cyt-C plays a vital function in programmed cell death [45, 46]. The release of Cyt-C from mitochondria into the cytoplasm initiates a cascade reaction, activates caspase signaling and regulates cell apoptosis. In addition, AIF exists in the inner mitochondrial membrane and is a factor that can induce caspase-independent apoptosis. In the process of apoptosis, AIF is transferred from mitochondria to the cytoplasm and then enters the nucleus, causing DNA agglutination and fragmentation in the nucleus, resulting in cell death [47].

To determine the underlying mechanism of realgar-induced APL cell death, we evaluated the effect of realgar on mitochondrial function. The expression of Bcl-2, Bax, Cyt-C, and AIF at the mRNA and protein levels was evaluated before and after exposure to various concentrations of realgar using immunofluorescence, western blotting, and qPCR experiments. Our results showed that Bcl-2 was decreased and Bax, Cyt-C, and AIF were increased at the mRNA level in a concentration-dependent manner after APL cells were treated with realgar (Figure 6). In addition, Bcl-2 was decreased, whereas Bax, Cyt-C, and AIF were increased at the protein level in a concentration-dependent manner after APL cells were treated with realgar (Figures 7 and 8). Therefore, our findings demonstrate that realgar induces APL cell death mainly through the abnormal regulation of mitochondrial function and that Bcl-2, Bax, Cyt-C, and AIF play essential roles in realgar-induced APL cell death. Interestingly, Xie et al. found that after realgar treatment, the expression of Caspase-3, Caspase-9 and Cyt-C in NB4 cells increased, thus initiating the caspase-dependent intrinsic apoptosis pathway [48].

Qi et al. studied the differential proteome after realgar was used to interfere with the APL retinoic acid-resistant cell line NB4-R1 [49, 50]. A total of 21 differentially expressed proteins were screened, of which 5 proteins were downregulated and 16 proteins were upregulated. Mass spectrometry showed that the differentially expressed proteins SET (I2PP2A), RPP0, RPP2, PCBP1, ACTB, HMGB1, PHB, RhoGDI2 and elFSA1 were closely related to the proliferation and apoptosis of APL cells. Wang et al. discovered changes in the gene expression profile after realgar acted on NB4 cells and found that the altered expression of PSMC2, PSMD1 and ITGB1 genes in particular may play an important role in the apoptosis and differentiation of NB4 cells [51, 52]. The interaction between the realgar-induced apoptosis effect and genomics and proteomics needs to be further investigated.

**Figure 9:**
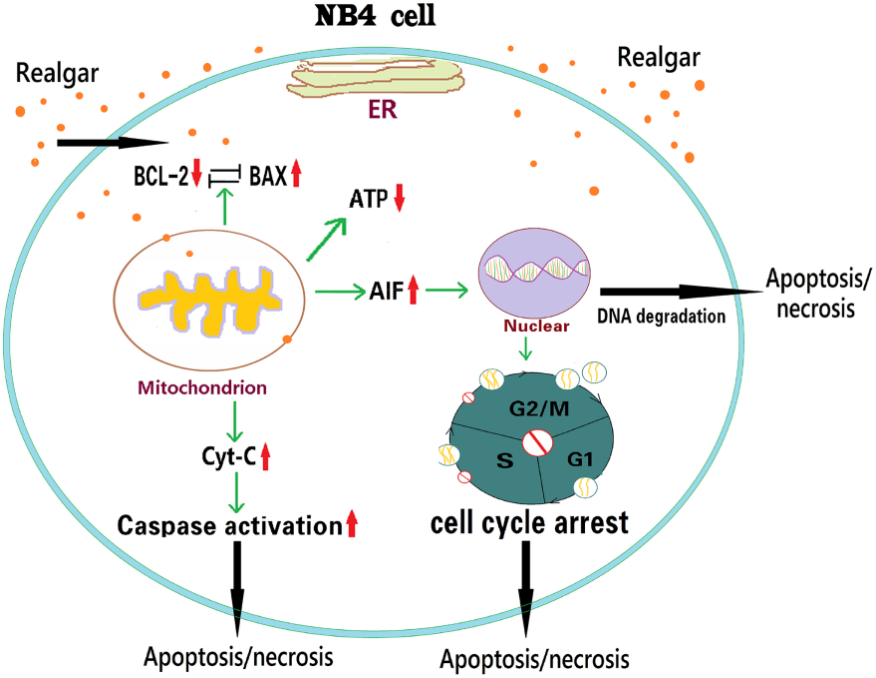
Schematic illustration of how realgar induces APL cell death via the Bcl-2/Bax/Cyt-C/AIF signaling pathway.

As shown in Figure 9, APL cells exposed to realgar reduced ATP production, destroyed the nucleus, and induced APL cell cycle arrest at the S and G2/M phases. In addition, realgar also decreased Bcl-2 expression while increasing Bax, Cyt-C, and AIF expression at the mRNA and protein levels. Our findings indicate that Bcl-2, Bax, Cyt-C, and AIF might play an essential role in realgar-mediated APL cell death.

## CONCLUSION

Overall, our results demonstrate that realgar can effectively induce APL (NB4) cell death *in vitro* in a dose- and time-dependent manner. Realgar treatment of APL cells can reduce ATP production, damage the nucleus, and induce APL cell cycle arrest at the S and G2/M phases. In addition, it can also disrupt mitochondrial homeostasis by decreasing Bcl-2 expression and increasing Bax, Cyt-C, and AIF expression at the mRNA and protein levels. Therefore, Bcl-2, Bax, Cyt-C, and AIF may play essential roles in APL cell death. Our findings provide new insight into realgar-induced APL cell death involved in mitochondria-mediated apoptosis, and indicate that realgar may be a potential therapeutic for APL in clinical practice. Animal studies should be performed next to confirm the therapeutic effects of ZnO NPs on MM.

## Supplementary Materials

Supplementary Figure 1
