# Supplementary material for: Realgar (As4S4), a traditional Chinese medicine, induces acute promyelocytic leukemia cell death via the Bcl-2/Bax/Cyt-C/AIF signaling pathway in vitro
Source: Aging (Albany NY). 2022 Sep 12;14(17):7109–25. doi: 10.18632/aging.204281 (PMC9512515; doi:10.18632/aging.204281)
Supplement: Supplementary Figure 1 [file aging-14-204281-s001.pdf]

SUPPLEMENTARY FIGURE

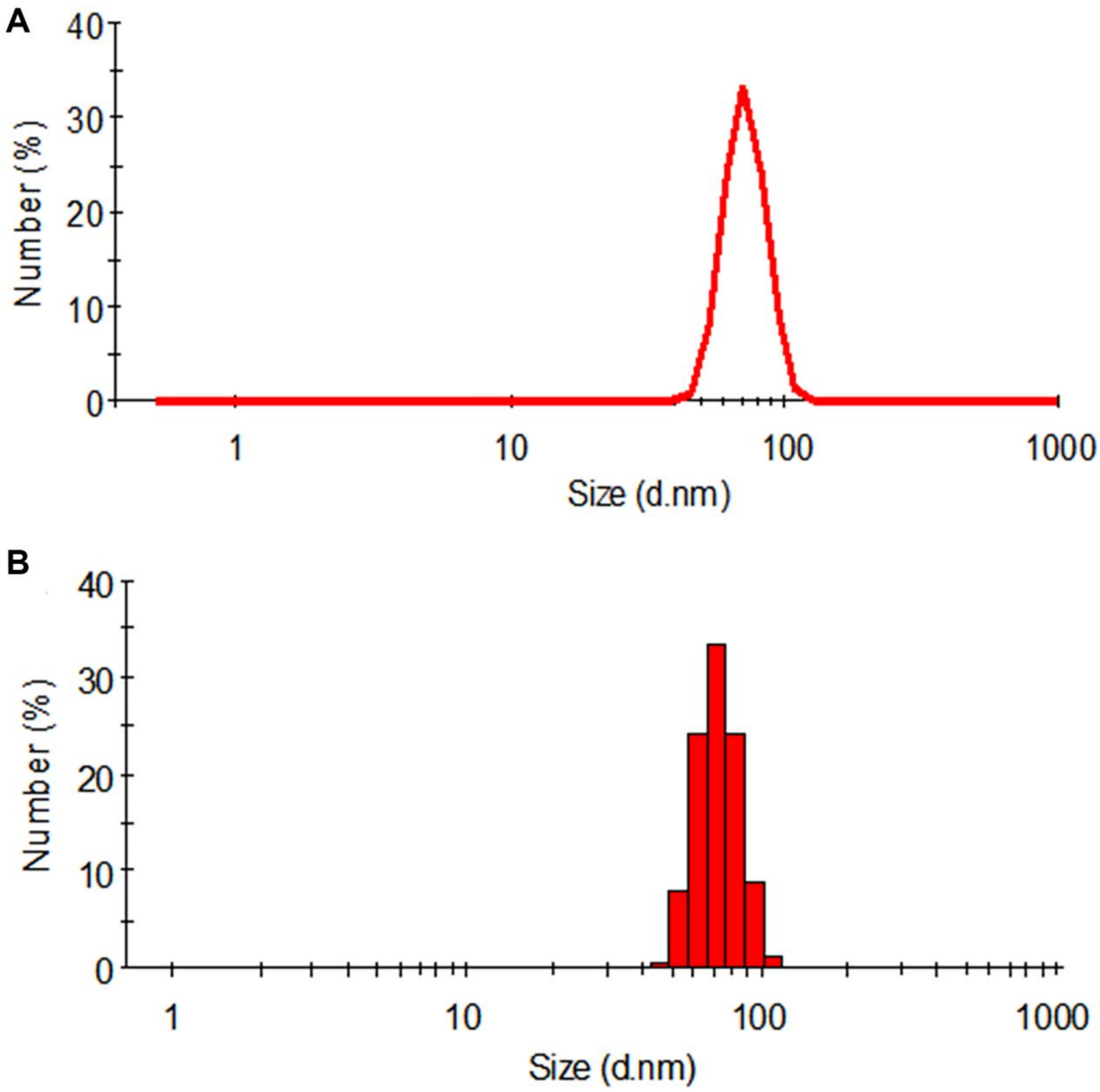

Supplementary Figure 1. Characterization of realgar. (**A**) DLS of realgar. (**B**) Size distribution of realgar.
